# Fetal lung MRI and features predicting post-natal outcome: a scoping review of the current literature

**DOI:** 10.1259/bjr.20220344

**Published:** 2023-06-06

**Authors:** Elspeth Whitby, Trevor Gaunt

**Affiliations:** 1 University of Sheffield and Sheffield Teaching Hospitals NHS foundation Trust, England, United Kingdom; 2 Department of Maternal and Fetal Medicine, University College London, London, United Kingdom; 3 University College London Hospitals NHS Foundation Trust, London, United Kingdom

## Abstract

The outcome for infants with fetal lung pathologies not only depends on the nature of the pathology, but the impact it has on the developing lungs. The main prognostic factor is the degree of pulmonary hypoplasia, but this is not detectable pre-natally. Imaging techniques aim to simulate these features with a variety of surrogate measurements, including lung volume and MRI signal intensity. Despite the complexity of the various research studies and lack of consistent methodology, this scoping review aims to summarise current applications, and promising techniques requiring further investigation.

## Introduction

The outcome for infants with fetal lung pathologies not only depends on the nature of the pathology but also on the impact the pathology has on the developing lungs. Over the years, there have been numerous studies aiming to establish imaging factors that predict outcome and whilst many have shown promising results the outcome is still highly variable. The main predicting factor is the degree of pulmonary hypoplasia. Unfortunately, this is not just related to lung volumes but also the quality of the lung that is present. Pulmonary hypoplasia is defined as reduced lung/body weight ratio at a macroscopic level, and alveolar underdevelopment on a histological level.^1,2^ This information is not available pre-natally, and hence the aim of fetal MRI is to simulate these features with surrogate measurements of the lung and body, and attempts to characterise the microscopic architecture of the lung *in utero*.^3^

The studies that have looked at detecting hypoplasia have based their information on lung size,^4–8^ lung signal intensities and signal characteristics.^9^ There are a few studies that have looked at signal changes in normal lungs^10,11^ and others that have compared normal groups to pathological groups. As the lungs are continually developing *in utero,* the normal will depend on the gestational age of the fetus. As with all fetal imaging parameters, the gestational age is hugely important and whilst studies do take the gestational age into account, many place the cohort into trimesters, while others round to the nearest gestation week making comparisons between studies difficult. As the fetus develops, the range of all measurements increases. Therefore use of a mean for each gestational age does not take into account the normal variation seen, especially if the fetus has additional pathology, *i.e.* small for gestational age. A few studies have tried to address this issue by comparing the ratio of fetal lung volumes to the total fetal body volume,^4^ others have compared lung signal to signal from amniotic fluid, liver^12–14^ or spinal fluid^15^ on the same image. Despite the complexity of each different study and lack of consistent methodology, this scoping review aims to summarize what is currently known and what looks promising but needs further investigation.

## Methods

Where necessary, the PRISMA extension for scoping reviews (PRISMA-ScR) protocol was used to guide this unregistered scoping review. The literature review was performed by one investigator (TG) within the bibliographic database of MEDLINE (PubMed), using pre-specified research terms relating to “fetal”, “prenatal”, “MRI”, “lung”, “postnatal” and “function” (see search strategy, supplementary material).

The latest search was performed on November 22, 2021. There were no pre-defined date limits or restrictions on sample size. Inclusion criteria were for peer reviewed papers in English. Articles not utilising fetal MRI were excluded. Case reports, review articles and opinion pieces were excluded, but their reference lists were interrogated for any further references. Articles regarding the cardiovascular system were also excluded. Gray literature was searched by using the Google search engine and “Google Scholar” database for organisational or societal documents describing the use of MRI in the assessment of the fetal lungs.

In order to demonstrate the full scope of MRI in fetal lung assessment, no exclusions were made on the basis of MRI sequence, pathology or outcome. A bias and quality assessment was not performed owing to the expected heterogeneity and small number of publications relating to intrinsic MRI assessment of the lung and post-natal outcome. We did not intend to reject any studies on this basis at scoping stage. For these reasons, a descriptive review of our findings was intended from the beginning.

## Results

**Figure 1.**
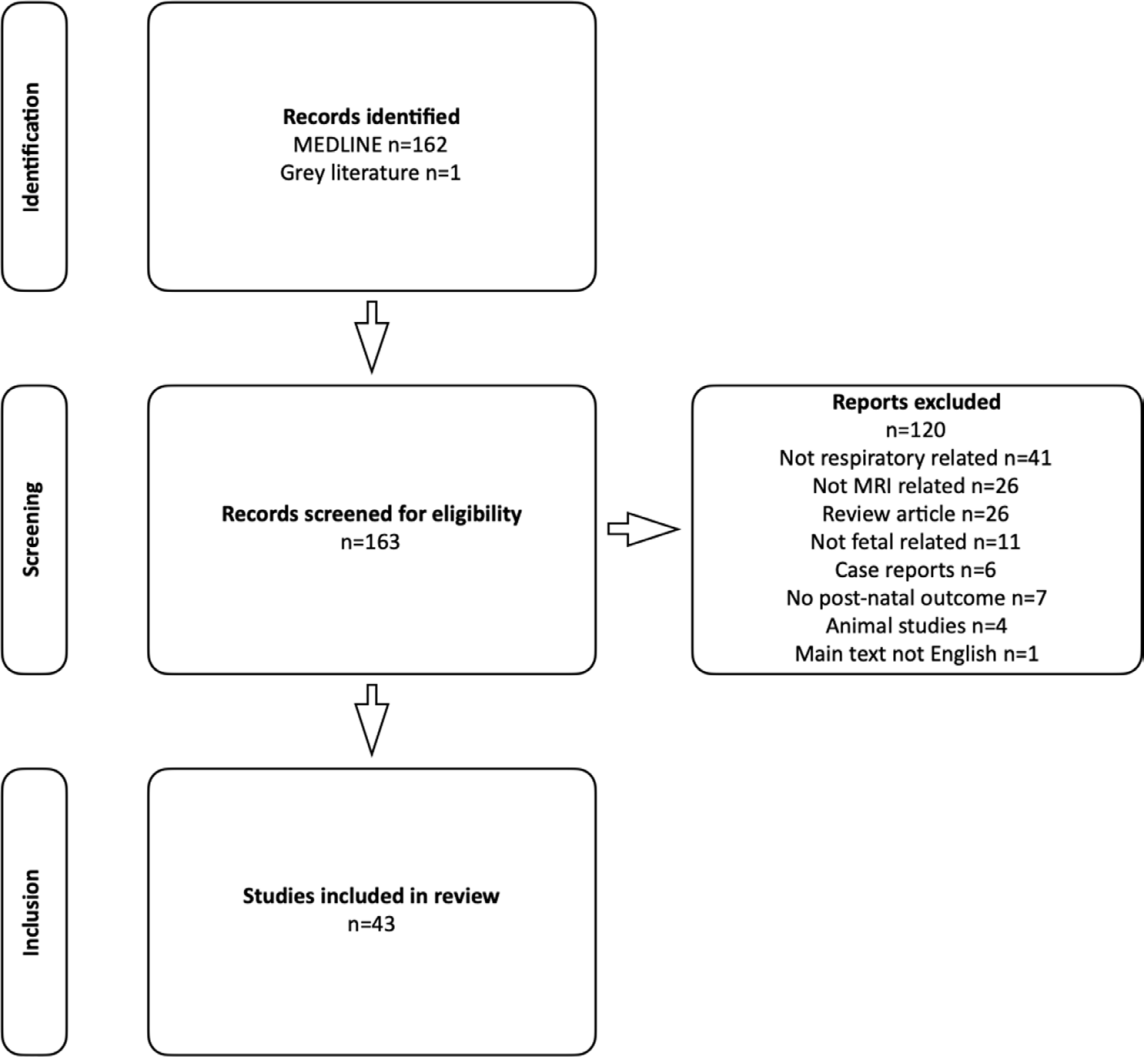
Exclusion tree demonstrating process of identification, screening and inclusion of studies for review

163 abstracts and titles were reviewed, with 43 articles meeting the inclusion criteria and 120 excluded (Figure 1). All articles were clinical in nature aimed at either clinical radiologists or fetal medicine experts. By far, the most common indication for fetal MRI was congenital diaphragmatic hernia (CDH), with fewer related to congenital lung abnormalities, high airway obstruction, genitourinary, cardiac and lymphatic abnormalities. The vast majority related to lung volumetry, with others assessing lung T2 and diffusion-weighted imaging (DWI) signal intensity, and a few regarding anatomy and morphology. The most frequently reported outcome end point was post-natal survival, followed by the need for extra corporal membrane oxygenation (ECMO), intensive care stay duration, oxygen dependancy, the need for supplemental feeds, persistent pulmonary hypertension, and lung maturation demonstrated at autopsy.

Pre-natal MRI features and post-natal outcomes could be classified into several broad groups detailed below, with MRI features reported in isolation or in combination. The most clinically relevant articles are expanded upon.

Observed total fetal lung volumes (TFLVs) were invariably calculated using free-drawn regions of interest (ROIs) around lung tissue on consecutive slices of fast *T*_2_ weighted sequences. Lung area on each slice was totaled and multiplied by slice thickness to give a volume. Observed lung volume was then expressed as a ratio of that expected for gestational age according to age-matched controls,^16^ age-related normograms^17^ or based on expected lung volumes derived from total fetal body volume^4^ (observed/expected ratio).

Lung volumes measured on fetal MRI have been shown to correlate well with lung area measurements performed on two-dimensional (2D) ultrasound.^18^

Moreover, o/e TFLV ratio has been shown to more accurately predict clinical outcome and severity of pulmonary hypoplasia, compared to observed/expected lung-head ratio (o/e LHR) on pre-natal ultrasound, when there is discordance between the two techniques.^19^

Weidner et al^20^ expressed TFLV as a ratio of total fetal body volume, arguing that the use of an internal control accounts for *individual* fetus development, rather than comparing against reference healthy controls. The group found FLV/FBV ratio was able to accurately predict neonatal survival and ECMO requirement in infants with CDH (AUC >0.8).

An in-depth demonstration of each specific measurement is beyond the scope of this review. However, each is well illustrated in the studies described below.

## Lung volume

### Survival

#### Congential diaphragmatic hernia

The majority of references detailed survival rates in fetuses with left CDH (LCDH), most including confounding factors such as intrathoracic liver herniation^21,22^ and stomach position within the thoracic cavity.^23^

Jani et al^5^ studied outcome in a total of 148 fetuses with isolated CDH (LCDH 127, RCDH 21). TFLV had been calculated on T2 HASTE sequences in a subpopulation of 76 fetuses who had undergone MRI on a 1.5 Tesla (1.5T) machine; the laterality of the hernia, however had not been described in this subpopulation. There was a direct correlation between higher o/e TFLV based on calculated fetal body weight (FBW), and higher survival rates. Survival rate increased from 12% in those with intrathoracic liver herniation and an o/e TFLV of ≤25%, to more than 70% for an o/e TFLV of ≥46%. The presence of liver herniation was a large confounding factor, with survival rate almost three times higher at 40% in fetuses with an o/e TFLV ≤25% and no liver herniation.

Brown et al^8^ described a predictive mortality algorithm in 41 fetuses, combining observed-to-expected TFLV based on gestational age related nomograms,^17^ intrathoracic position of the stomach, and post-natal ECMO requirement. O/e TFLV was significantly associated with mortality, with a mean o/e TFLV of 43% in survivors and 24% in non-survivors (*p* = 0.007). Stomach position in the chest was also associated with mortality (*p* = 0.049).

Right CDH (RCDH) is rare compared to LCDH, accounting only 15–20% of cases and a poorer overall survival rate.^24^ Russo et al^16^ found that o/e LHR and o/e TFLV based on FBW were similar in 86 fetuses with RCDH. Overall survival rate of expectantly managed fetuses was 46%. Survival rate was 15% in those with o/e LHR <45% and 61% for o/eLHR ≥ 45% (*p* = 0.001). No fetuses with o/eLHR <30% survived.

In summary, mortality in RCDH is associated with greater lung volumes than LCDH, suggesting it may be a distinct disease entity with unique pathophysiology. Intrathoracic stomach and liver positioning confer a worse prognosis in LCDH, compared to those fetuses without stomach and liver herniation.

#### Omphalocoele

Danzer et al studied post-natal survival in fetuses with giant omphalocoele based on o/e TFLV ratios according to gestational nomograms. Based on previous observations,^7^ o/e TFLVs were grouped into ≤50% and >50% to stratify for severity of pulmonary hypoplasia. Fetuses with o/e TFLV ≤50% were further grouped into 25-50% and <25%, the latter regarded as severe pulmonary hypoplasia. O/e TFLV ratios were calculated according to Meyers normograms for all 67 fetuses^25^ and according to Rypens normograms^17^ in a subpopulation of 49 fetsuses (18 fetuses fell outside the GA range for Rypens).^6^ Using Meyers’ normograms, they found a significant inverse relationship between lung volume and risk of neonatal morbidity; survival with severe pulmonary hypoplasia was 60%, compared to 92% with moderate, and 96% with mild hypoplasia (*p* = 0.04). A similar but non-significant trend was observed using Rypens’ nomograms.

#### Congenital high airway obstruction (CHAOS)

CHAOS is defined as any fetal abnormality that obstructs the larynx or trachea. Fluid produced within the fetal lungs is trapped by the obstruction, and the lungs become abnormally distended. Mong et al^26^ compared TFLVs in 10 cases to normal values for gestational age.^17^ MR lung volumes were elevated in 9 of the 10 fetuses, measuring between 3.8 and 12.7 standard deviations above the gestational age mean for normal fetal lung volume. However, seven of these cases were either terminated or died *in utero,* meaning it was not possible to deduce any relationship between lung volume and post natal survival.

Conversely, and despite airway obstruction, pulmonary *hypoplasia* has been reported in neonates born with a giant anterior cervical teratoma. Liechty et al showed 3 of 11 fetuses with giant anterior cervical teratoma developed and succumbed to severe pulmonary hyopplasia. At post-mortem, the lungs had been retracted into the thoracic apices and compressed by the cervical mass following extreme neck hyperextension. The airways had remained patent, further supporting a mechanical or compressive aetiology.^27^ Wolfe et al ^28^ used o/e TFLVs according to gestational nomograms^17^ to predict survival in 12 fetuses with CHAOS secondary to cervical teratoma. A TFLV greater than one standard deviation below the median for gestational age had a positive-predictive value of 100% for lethal pulmonary hypoplasia. Moreover, a TFLV *less* than one standard deviation below the median was uniformly associated with survival (100% negative-predictive value).^28^ These values appear very different to those in cases of CDH.

#### Genitourinary abnormalities and oligohydramnios

Zaretsky et al^29^ investigated TFLV–gestational age ratio, for predicting outcome in fetuses with genitourinary abnormalities. This was determined by dividing calculated lung volume by gestational age. A TFLV–gestational age ratio of 90% had a sensitivity and specificity of 85.7% and 83.3%, respectively, for predicting neonatal death but only after 26 weeks gestational age. Interestingly, there was no significant difference in prediction of survival between TFLV–gestational age ratio and the presence or absence of oligohydramnios in fetuses with genitourinary abnormalities.

Messerschmidt et al^30^ demonstrated significantly lower o/e TFLV in non-surviving fetuses with pre-term pre-mature rupture of membranes (pPROM) and oligohydramnios. Using 109 normal fetuses as gestational age-matched controls, and 40 cases of pPROM, non-survivors had a mean o/e TFLV of 73%. There were no survivors with an o/e of <60%, the sensitivity, specificity and accuracy of which being 80%, 86% and 85%.

### ECMO

#### Congenital diaphragmatic hernia

In addition to survival, many articles regarding CDH used lung volumes to predict the need for ECMO and sustained respiratory support.

Kilian et al^31^ studied the predictive need for ECMO therapy in 28 infants with CDH based on “relative” fetal lung volume (rFLV akin to o/e ratio used in other studies). They found no neonate with a prenatal MRI rFLV > 44% needed ECMO therapy.

### Pulmonary hypertension

#### Congenital diaphragmatic hernia

Several groups have investigated whether lung volumes correlate with persistent pulmonary hypertension in CDH,^32^ yielding conflicting results. Kim et al^19^ found persistent pulmonary hypertension in 13.3% of those with mild-, 50% of moderate-, and 100% of patients with severe pulmonary hypoplasia according to o/e TFLV (<25%, 25–35% and >35% o/e TFLV, respectively).

Coughlin et al^32^ identified persistent pulmonary hypertension in 58% of surviving neonates with CDH, but no significant difference in o/e TFLV between them (mean 21% o/e TFLV in both groups). However, the same group later demonstrated a relationship between o/e TFLV and severity of persistent pulmonary hypertension in a separate study.^33^

### Sustained respiratory and nutritional support

#### Congenital diaphragmatic hernia

Multiple studies in CDH concluded that fetuses with moderately and severely reduced lung volumes were associated with a greater length of stay in intensive care, and a greater need for invasive respiratory support, home oxygen, and nutritional support. Abbas et al^34^ investigated whether post-natal pulmonary gas exchange parameters correlated with CDH patient survival. They calculated o/e TFLV ratio according to gestational age-related nomograms in 57 fetuses with isolated CDH. Although initial PaCO_2_ in non-survivors was higher, infants with persistent post-resuscitation hypercarbia had a worse prognosis than those who resuscitated to a normal. However, MRI-derived o/e TFLV did not strongly correlate with PaCO_2_ levels. This suggests that while MRI measured lung volumes are a good predictor of overall outcome, this cannot necessarily be ascribed to gas exchange function.

#### Lung masses

Zamora et al evaluated whether lung mass volume ratio (lung mass volume cm^3^ / head circumference cm, LMVR), o/e TFLV, and lesion-to-lung volume ratio (LLV) could predict perinatal outcomes and lung-related morbidity in 113 fetuses with congenital lung masses (CLMs). The specific types of lung mass were not described. In fetuses >26 weeks gestation each measure correlated with neonatal respiratory distress, intubation, and NICU admission duration. An o/e TFLV of <75% was predictive of a worse post-natal course, with 57% sensitivity and 80% specificity.^35^

## Signal intensity

Lung signal intensity ratios were measured by dividing the arbitrary signal intensity value from an ROI drawn in the fetal lung, by another ROI drawn around a target organ. The majority of references examined lung T2 signal intensity as a marker of prognosis, in fetuses with CDH. Others measured diffusion weighted imaging (DWI) signal intensity and apparent diffusion coefficient (ADC) values.

### Liver–lung signal intensity ratio

Yamamoto et al^13^ compared liver–lung signal intensity ratio (LLSIR) in 30 fetuses with LCDH against a population of healthy controls. ROIs were drawn around the right lung in all cases. Fetuses were allocated into a poor prognosis group if the case ended in post-natal death or required ECMO. In the control group, LLSIR increased with gestational age, whereas in the CDH group, especially in the poor prognosis group, LLSIR did not significantly increase with gestational age.

Yokoi et al^12^ found a lower LLSIR conferred a poorer prognosis in a study of 25 fetuses with CDH. In fetuses where ipsilateral lung was not detectable at the thoracic apex, post-natal death before 2 days was more likely if the LLSIR was <2. All patients with no ipsilateral lung detectable by MRI required inhaled nitrous oxide, compared to only 27% of those where ipsilateral lung was detectable. Similarly, Oka et al^14^ studied LLSIR in 110 fetuses who underwent MRI for a variety of indications (22 CDH and 2 pleural effusions). Using ROC curve analysis, they found fetuses with a LLSIR <2.0 were more likely to develop severe respiratory distress with a sensitivity of 100% (95% CI 52–100%) and a specificity of 73% (95% CI 54–88%). However, their data also suggest that half of the cases *without* respiratory distress also had LLSIR below 2.0. LLSIR therefore needs further research before it can be used in clinical practice.

Ballassy et al^36^ examined both T1 and T2 LLSIR in 25 fetuses with LCDH, compared with 91 gestational age-matched controls. LLSIRs in fetuses with LCDH were significantly higher in both lungs on *T*_1_ weighted images and significantly lower on *T*_2_ weighted images, compared with normal controls. LLSIR increased on *T*_2_ weighted imaging and decreased on *T*_1_ weighted imaging during gestation. No significant differences in LLSIR between the two lungs were seen, and in contrast to Yokoj et al, LLSIR was not significantly different in the primarily unaffected contralateral lungs of survivors and non-survivors, again illustrating the need for further large studies.

### Lung–spinal fluid signal intensity ratio

Terui et al^15^ retrospectively compared lung–spinal fluid (L/SF) signal intensity in 12 fetuses with CDH (10 LCDH, 2 RCDH). ROIs were drawn around contralateral lung tissue and within the spinal CSF. The mean signal intensity of the contralateral lung was divided by that of spinal fluid for each slice. This was repeated for all lung sections through the chest, and the mean value for the L/SF ratio was calculated. L/SF of normal controls were referred to in a previous paper from the same group.^37^ L/SF were significantly larger in survivors (*n* = 8, L/SF ratio 0.815) compared with non-survivors (*n* = 4, L/SF ratio 0.614, *p* < 0.05). In survivors, L/SF significantly correlated with duration of tracheal intubation (rs ¼ 0.938, *p* < 0.01).

### Other signal intensity ratios and ADC values

In a recent attempt to clarify the role of various signal intensity ratios, Dütemeyer et al compared the value of lung volume measurements and lung signal intensity ratio using liver, amniotic fluid, muscle or spinal fluid to create several ratios against neonatal outcome.^38^ In 75 conservatively managed CDH fetuses, o/e TFLV, LLSIR, and lung muscle signal intensity ratio all predicted post-natal survival, although o/e TFLV was better with an AUC of 0.938 compared to 0.688 and 0.663 for LLSIR and lung muscle signal intensity ratio respectively.

Finally, Cannie et al^9^ investigated ADC values in 93 normal fetal lungs, and 14 with CDH. All visible areas of each lung on all slices were delineated, and combined to create one 3D ROI per lung. This was performed on each DWI sequences performed at six different b-values, and an average calculated (ADCavg) for each lung. Separate ADC average values were calculated for three low b-values (*b* = 0, 100 and 250 s/mm^2^; ADClow) and three high b-values (*b* = 500, 750 and 1000 s/mm2; ADChigh). In fetuses with normal lungs, there was a negative correlation between ADChigh and a positive correlation with ADClow and gestational age. 4 of the 14 fetuses with CDH did not survive meaning the non-survivor subpopulation was too small for statistical analysis. However, for the ipsilateral lung, the o/e ADClow in these fetuses tended to be higher, suggestive of a greater degree of diffusion restriction, and lung tissue with a greater density. Furthermore, ADClow was independent of other predictors of outcome such as lung volume, and hence could potentially be used as an additional biomarker in predicting post-natal survival; a lower o/e ADClow in the ipsilateral lung might be predictive of lung hypoplasia.

## Anatomy and morphology

### Congenital diaphragmatic hernia

A small number of studies examined the effect of other anatomical and morphological features on survival in the context of CDH. Spaggiari et al^39^ investigated the prognostic significance of the presence of a hernia sac in 70 cases of isolated CDH. They found 32.7% of neonates *without* a hernia sac did not survive. Only 5.6% of those *with* a hernia sac did not survive. Those with a sac had a significantly higher o/e TFLV, suggesting its presence may confer a more favourable prognosis.

### Congenital diaphragmatic hernia sac contents

The presence of herniated liver in LCDH (“liver up”) has long been established as a feature of poor prognosis.^23,40–43^ Victoria et al^44^ showed survival in fetuses with isolated LCDH was 45% with “liver up” compared to 94% with “liver down” in a cohort of 85 isolated cases. Worley et al^45^ compared neonatal outcome with the percentage of the fetal thorax occupied by lung, liver, and other abdominal organs in 15 fetuses with isolated CDH (11 LCDH, 4 RCDH). They measured the areas of total thorax (excluding spine), both lungs, liver, and herniated gastrointestinal tract, on axial slices through the thorax and calculated the individual volumes. Liver herniation was found in 93%. If greater than or equal to 20% of the thorax was occupied by liver, the neonatal mortality was 86% (95% CI, 42–100%) compared with 13% (95% CI, 0–53%) when less 20% of the thorax was occupied.

### Congenital diaphragmatic hernia laterality

The laterality of the hernia is also important. It is well established that right-sided CDH had a poorer prognosis than left-sided CDH, however this is currently being challenged. The findings of recent retrospective reviews suggest that the mortality is not different but the morbidity is higher with right-sided CDH. Hedrick et al^46^ studied 22 RCDH diagnosed in the antenatal period and 5 cases diagnosed in the post-natal period. They demonstrated that 12 of 23 surviving fetuses (52%) with a right-sided CDH containing the liver required ECMO, with a 75% survival rate.

Collin et al^47^ found the only increased morbidity was the rate of recurrent herniation in a group of 49 cases undergoing surgical repair (10 RCDH, 39 LCDH). However, Partridge et al^48^ in a study of 330 cases (274 left-sided and 56 right-sided) found that right-sided hernias had increased morbidity with a greater likelihood of needing a tracheotomy and vasodilator therapies.

### Mediastinal shift angle

Romiti et al^49^ measured the ‘mediastinal shift angle’ in 28 fetuses with pre-natally diagnosed isolated left CDH. The best ROC cut-off value with the highest discriminatory power to correctly classify survival, maximizing sensitivity and specificity, was 39.1 degrees, with a sensitivity 71.4%, specificity of 80.9%, positive-predictive value of 55.6%, negative-predictive value of 89.5%, and accuracy of 78.6%. They do not present any data on lung volume, o/e LHR in this cohort, so it is difficult to assess the value of the mediastinal angle compared to other variables.

### Nuchal translucency

Finally, Spaggiari et al^50^ examined the association between increased nuchal translucency thickness (NT) in the first trimester and perinatal outcome in 71 live born infants with isolated CDH; all fetuses with concomitant chromosomal abnormalities or malformations were excluded. The fetal NT was above the 95th percentile in 9 of the 71 cases. Neonatal death occurred in 7/9 (78%) cases with enlarged NT, compared with 24/62 (38%) with normal NT (*p* = 0.035). They concluded that enlarged NT was significantly associated with pre-natal features of intrathoracic compression, and postulate that NT in the first trimester is as a strong prognostic marker for post-natal survival in isolated CDH, albeit in a small population.

## Discussion

In this article, we have found a small number of research studies exploring the ability of fetal MRI to predict post-natal respiratory morbidity and mortality. All studies were performed at 1.5T. The vast majority of these studies, and those with the greatest sample population, concerned cases of fetuses with CDH. A minority concerned giant omphalocele, congenital high airways obstruction syndrome and lung masses. Most were deemed feasibility studies.

The most common technique for predicting outcome was the measurement of lung volumes on *T*_2_ weighted fast spin echo sequences, expressed as an o/e TFLV ratio. The o/e TFLV ratio was most frequently derived from gestational age-related nomograms, whereas a smaller number were derived from measured TFBV. Studies of LCDH were more common than RCDH, and the presence of the liver within the hernia sac conferred a worse prognosis. RCDH demonstrated greater post-natal morbidity and mortality than LCDH for a similar o/e lung volume.

The study of lung signal intensity as a ratio of liver, muscle and CSF comprised a small minority of articles. The techniques used were heterogeneous amongst the different articles, with small study populations. Again, the most frequently studied pathology was CDH. Fetal lung T2 signal increases throughout gestation,^10,11^ and fetuses with poorer outcome demonstrated smaller lung signal intensity ratios compared to normal controls. However, interobserver reliability was shown to be only moderate at best.^10^ One study investigated the benefit of performing DWI sequences to assess the fetal lungs, finding that lower ADC values at low b-values might be predictive of poorer outcome in a small number of fetuses with CDH.^9^

This review has some limitations. Our search strategy included only the most popular research databases believed to provide the greatest number of relevant articles. A search of the gray literatures returned no additional studies after exclusion. A future systematic review should consider all databases and could attempt a meta-analysis to further determine specific and MRI characteristics, ideally with cut-off values, predictive of survival and the need for post-natal respiratory support. An in-depth reproducibility analysis amongst various techniques could also be performed. Our review has not included the whole gamut of MRI techniques available as some, such as magnetic resonance spectroscopy, were only performed in normal fetuses, and not studied in relation to respiratory outcome. However, given the constant improvement in MRI hardware, software, and post-processing data manipulation,^51^ these techniques may be accessible in the future.

Future directions for fetal MRI and prognostication are exciting. Bluml et al^52^ investigated the feasibility of using magnetic resonance spectroscopy to quantify the lipid content of amniotic fluid in normal fetuses as a function of the gestational age, and therefore a potential surrogate marker for lung maturation. The lipid-to-water ratio remained steady until after 36 GW, at which point it increased exponentially. The authors postulate that this could potentially replace invasive sampling via amniocentesis if validated in a larger study.

Khen-Dunlop et al have recently studied the blood oxygen level dependent (BOLD) effect in the fetal lungs.^53^ After inducing maternal hyperoxia for 5 min, a significant BOLD response was observed in the fetal lungs, demonstrating oxygen uptake. The BOLD effect could be studied in fetuses with pathological lungs, and potentially add useful information for pre-natal counselling and treatment planning.

Fetal scanning at 3T is not yet common place, however is becoming more mainstream and attractive owing to signal incrteases at higher magnetic field strengths.^54,55^ Afacan et al established a range of normal values for ADC in the lungs of normal fetuses at 3T. ADC values showed a strong association with gestational age increasing dramatically between 16 and 27 weeks and plateau around 27 weeks.^56^ This poses a challenge however as main magnetic field, and radiofrequency magnetic field, inhomogeneity artifacts increase at higher field strengths.

In current practice, fetal MRI makes use of spin echo and gradient echo sequences to acquire 2D data stacks of the fetus. The most challenging obstacle is fetal movement, which leads to anatomical misregistration and signal corruption. Currently therefore, fetal MRI relies on acquiring data during fetal quiescence. This is unpredictable and often necessitates sequence repetition, contributing to increased scan duration and maternal fatigue. Uus et al recently showed post-processed deformable slice-to-volume registration reconstruction provided high resolution 3D volume data sets derived from multiple 2D stacks in the fetal body.^57^ If clinically validated, combining multiple 2D stacks to create a single 3D reconstructed data set would allow image manipulation in multiple planes, improved spatial resolution, and a reduction in scan duration.

In conclusion, current prognostication in fetal lung MRI is largely reliant on volumetric assessment of the fetal lungs using 2D fast spin echo sequences in a handful of pathologies. If emerging techniques can be employed and validated in pathological lungs, accurate, artifact-free, functional assessment of the fetal lungs at higher field strengths, could be feasible for predicting post-natal respiratory outcome.
